# Effects of Chinese provincial CDCs WeChat official account article features on user engagement during the COVID-19 pandemic

**DOI:** 10.7189/jogh.13.06005

**Published:** 2023-04-14

**Authors:** Mingjuan Yin, Senke Chen, Xueyan Pan, Caixia Lu, Xiaojie Lin, Mingwei Wang, Jindong Ni

**Affiliations:** School of Public Health, Guangdong Medical University, Dongguan, China

## Abstract

**Background:**

WeChat has become a potent medium for disseminating public health information, especially during the coronavirus disease 2019 (COVID-19) pandemic. WeChat is important for public health organizations when considering users’ information needs and preferences to further explore factors that affect user engagement.

**Methods:**

We collected data from WeChat official accounts (WOAs) of the Chinese provincial Center for Disease Control and Prevention (CDC) to identify factors affecting and predicting the behavior of user engagement as measured by the level of reading and re-sharing during different phases of the COVID-19 pandemic between January 1, 2019, and December 31, 2020. We used multiple logistic regression analyses to identify features of articles with higher reading and re-sharing levels from 31 Chinese provincial CDCs. We developed a nomogram to predict the effect on user engagement.

**Results:**

We collected a total of 26 302 articles. Release position, title type, article content, article type, communication skills, marketing elements, article length, and video length were key determinants of user engagement. Although the feature patterns also varied between different pandemic stages, the article content, release position, and article type were still the most prominent features driving user engagement. Regarding article content, the COVID-19 pandemic report and guidance for public protection were more likely to obtain high-level reading (normalization: odds ratio (OR) = 12.340, 95% confidence interval (CI) = 9.357-16.274) and re-sharing (normalization: OR = 7.254, 95% CI = 5.554-9.473) than other contents throughout the pandemic. When we compared release position with secondary push, users who used main push were more likely to exhibit high-level reading and re-sharing during any period, especially during normalization (OR = 6.169, 95% CI = 5.554-6.851; OR = 4.230, 95% CI = 3.833-4.669). For article type, a combination of text, links and pictures was associated with a higher rate of reading (normalization: OR = 4.262, 95% CI = 3.509-5.176) and re-sharing level (normalization: OR = 4.480, 95% CI = 3.635-5.522) compared to text only. Simultaneously, the prediction model showed good discriminatory power and calibration.

**Conclusions:**

Discrepancies exist in article features between different pandemic stages. Public health agencies should make full use of official WOAs and consider the information needs and preferences of users in order to better carry out health education and health communication with the public when public health events occur.

Despite unprecedented efforts taken to halt it, the coronavirus disease 2019 (COVID-19) outbreak actively threatens global public health [1]. After 2021, the international COVID-19 pandemic took on a new dimension with new but low-virulent strains such as Omicron leading to mild clinical symptoms, making countries dynamically adjust their prevention and control measures [2]. However, due to higher infectivity, transmission, and immune escape ability, there is a need for the general population, especially the unvaccinated and immunodeficient subgroups, to get the latest COVID-19 information [2-5]. Therefore, effective risk communication between public health organizations and the public to minimize panic and offer both personal protection guidance and effective medical services was important during the early stages and at key turning points of dynamic changes of the pandemic [6]. Over the past decade, media landscapes have evolved significantly and engaged a wide audience, even reaching minority groups that have difficulties accessing health care interventions [7,8]. Moreover, there is increased attention to health and the frequency of public social media use because of pandemic-related quarantine and movement limitation measures. Consequently, social media has become a potent medium for instantaneously sharing public health information and engaging users, especially in face of a pandemic.

WeChat has grown rapidly in popularity and become the most popular information-sharing platform in China, with over 1.2 billion monthly active users worldwide as of the first quarter of 2020 [9,10]. Notably, 93% of residents log onto WeChat every day in first-tier cities of China [11]. Moreover, it was used approximately 78.7% more frequently following the COVID-19 outbreak [12]. WeChat users are spread quite evenly between different age groups, which is convenient for carrying out health education among the older population, since popular media such as Sina Weibo mainly focus on young people [13,14]. Because of its high popularity and multiple functions, an increasing number of official media outlets are sharing information on the WeChat platform. WeChat official accounts (WOAs) (a WeChat-specific module) are widely used as a dissemination and bidirectional communication channel for health information and interventions [15], making it necessary to determine the role of articles from the WeChat platform in meeting public needs during emergencies.

The Center for Disease Control and Prevention (CDC) is one of the first professional public health authorities that the public depends on for education, recommendations, and dissemination of factual information [16,17]. The official CDC account provides increased effective information disclosure and risk communication between the CDC itself and the public in a responsible manner [18], especially in the early stage of a public health emergency and key turning points of dynamic changes in the pandemic. However, thanks to its potential, health departments should further utilize social media [19]. Health departments must consider the users’ needs and preferences to further increase dissemination and user engagement, rather than just acting as unidirectional news outlets. The COVID-19 pandemic provided an opportunity for the CDCs to use article sharing features that the public pay attention to and successfully attract and engage users in health promotion.

Researchers have identified factors relating to government agency’ use of social media and public engagement during a public health emergency, but mainly involving Facebook and Twitter (which are frequently used abroad [20]), specific single accounts [21], or changes in number and content of articles [17,22]. Additionally, the primary period for data acquisition focused on the early stage of the COVID-19 pandemic [16,23]. However, growing evidence shows that the patterns of attractive article change between pandemic phases, for example, during the Zika pandemic [24]. It is unknown whether sentiment, article type, or other article features affect user engagement [17,20]. It is necessary to analyze these features of WOA information from public health organizations to determine significant details and engage with public health audiences, especially in different pandemic stages. We aimed to analyze the basic characteristics of article WOAs by Chinese provincial CDCs and identify the factors affecting and predicting the behavior of user engagement as measured by the level of reading and re-sharing during different phases of the COVID-19 pandemic. More optimized health communication strategies for government health agencies in response to public health emergencies will thus be provided.

## METHODS

### Study data collection

The element and engagement metrics for this study consisted of all articles posted by already existing WOAs of the Chinese provincial CDCs between January 1, 2019, to December 31, 2020. All data collection was conducted from February 10 to July 31, 2021. Provincial CDC WOAs published a total of 26 302 articles between 2019 and 2020, all of which we included in our analyses. All data are publicly available on WeChat.

### Variables and characteristics

Incorporating characteristics relevant to the COVID-19 pandemic, we developed a standardized questionnaire and established the coding norms to extract features based on the previous frame used by Zhang et al. [25]. A frame includes nine characteristics composed of push time, release position, title type, article content, article type, communication skills, marketing elements, article length, and video length. Based on this coding instrument, trained professional interviewers collected data through an online survey. SKC, CXL, and MWW resolved uncertainties regarding the answer choice by discussion following a full assessment. Detailed variable definitions are shown in Table S1 in the **Online Supplementary Document**.

### User engagement behaviors

WeChat engagement behavior is defined as users reacting to an article, such as reading, loving, and re-sharing. Due to the low level of thumbs-up behavior, we only included two dependent variables – reading and re-sharing level. Reading was defined as “How many people have read the article?” and re-sharing as “Readers rebroadcast them by simply retweeting them to a public space on WeChat, others can read articles being shared by ‘Discover>Top Stories>Wow’”. We gained “reads” or “Wow” options by clicking on “Subscriptions-articles” at the bottom of each article on the WeChat homepage.

### Statistical analysis

Independent variables, article type, communication skills, and marketing elements do not contain mutually exclusive categories in an article, so we reclassified and coded databased on the most common combination (>85%). Because combinations of marketing elements are scattered, we converted characteristics into quantities. The different stages of the COVID-19 pandemic were defined according to the daily cumulative confirmed cases data in China between January 22 to December 31, 2020 [26]. For outcome variables, we used the 75^th^ percentile as the cut-off point to categorize participants as high or low reading level and re-sharing level because the data were not normally distributed.

We generated descriptive statistics for characteristics of WOA articles to determine the frequency of each coding item. We used a χ^2^ test to determine the difference in categorical data and filter variables (*P* < 0.05). With low reading and re-sharing level category as a reference, we used binary logistic regression analysis to study the association between article characteristics (categorical independent variables) and user engagement to obtain the odd ratios (ORs) and corresponding 95% confidence intervals (CIs). We considered *P* < 0.05 (two-sided) as statistically significant.

Furthermore, we used significant variables in the logistic regression analysis as the final predictors to create a nomogram. We also used a receiver operator characteristic (ROC) curve to evaluate the discriminative performance of the nomogram model [27]. We used bootstrapping to perform internal validation of the original data to predict the accuracy of the nomogram model. The sum of each variable’s total score can be used to estimate the probability of high level of user engagement behaviors. We performed all statistical analyses using SPSS (version 25), Python (version 3.10.4), and R (version 4.2.1).

## RESULTS

### Characteristics of article features

After a manual search of provincial CDC-related keywords via the platform’s public search function, we found 31 Chinese provincial CDCs had opened WOAs for the dissemination of health knowledge. We coded a total of 26 302 articles (**Table 1**). Guangxi Province CDC had the highest proportion among the included articles, while the Xinjiang Province CDC had the lowest (n = 47, 0.2%). The most common code of release position, title type, article content, article type, communication skills, marketing elements, article length and video length were secondary push (53.4%), declarative sentence/ phrase (59.1%), content not related to COVID-19 (57.8%), text and pictures (60.6%), guidance/education/advice/appeal (68.7%), one marketing element (60.0%), <1000 words (61.6%), and no video (89.0%) (**Table 1**).

**Table 1:** Characteristics of the article features

Article features	Participants, n (%)
N (%)	26 302
**Stage of COVID-19 pandemic**	
Non-pandemic	7472 (28.4)
Outbreak	4152 (15.8)
Normalization	14 678 (55.8)
**Release position**	
Main push	14 055 (46.6)
Secondary push	12 247 (53.4)
**Title type**	
Declarative sentence/phrase	15 548 (59.1)
Exclamation/emphasis	4831 (18.4)
Question sentences	3310 (12.6)
Combinations of the above sentences	2613 (9.9)
**Is the content of the article related to COVID-19?**	
No	15 208 (57.8)
Yes	11 094 (42.2)
**Article type**	
Text only	1985 (7.5)
Picture only	1232 (4.7)
Text and pictures	15 946 (60.6)
Text, links and images	3823 (14.5)
Text, video and pictures	1331 (5.1)
Other	1985 (7.5)
**Communication skills**	
Guidance/education/advice/appeal	18 062 (68.7)
Positive emotional appeal	1782 (6.8)
Guidance/education/advice/appeal and Positive emotional appeal	1818 (6.9)
Guidance/education/advice/appeal and positive emotional appeal	1488 (5.9)
other	3152 (12.0)
**Number of marketing elements**	
0	7250 (27.6)
1	15 790 (60.0)
>1	3262 (12.4)
**Article length**	
<1000 words	16 200 (61.6)
1000-1499 words	5070 (19.3)
1500-2000 words	2171 (8.3)
>2000 words	2861 (10.9)
**Video length**	
None	23411 (89.0)
1-149s	1470 (5.6)
150-300s	746 (2.8)
>300s	675 (2.6)

s – seconds

**Figure 1:**
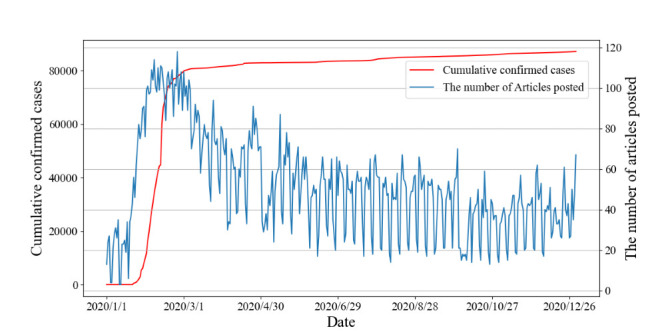
Longitudinal trends of WeChat official account articles and coronavirus disease official case counts.

After the COVID-19 outbreak, the number of articles increased approximately 2-fold as compared to the previous year. According to push time, we analyzed dynamic changes of WOA articles and official COVID-19 case counts. The number of articles posted by WeChat and the progress of the COVID-19 pandemic are nearly synchronized (**Figure 1**). According to longitudinal trends, we defined March 1, 2020, as an inflection point toward normalization of the pandemic, after which the number of articles gradually decreased.

### Analysis of features affecting the user engagement behaviors during the COVID-19 pandemic

**Figure 2:**
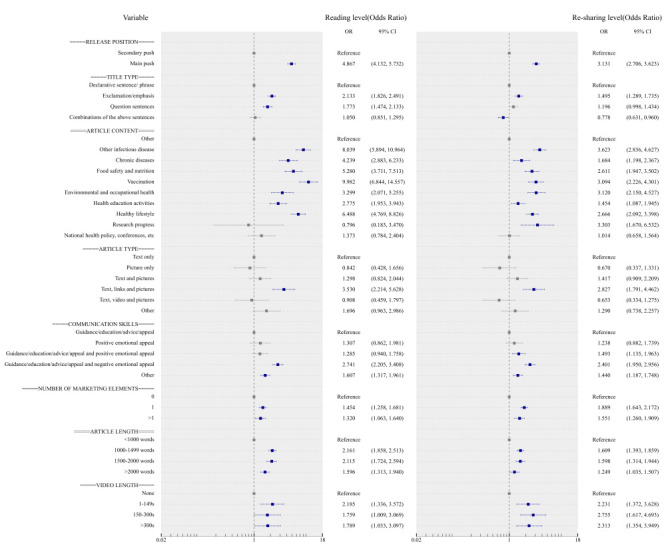
Multivariate logistic regression analysis of reading level and re-sharing level during non-pandemic period. s – seconds.

Multivariable logistic regression showed that prior to the pandemic release position, title type, article content, article type, communication skills, marketing elements, article length and video length contributed significantly to explaining the level of engagement (**Figure 2**). Concerning release position, when compared with secondary push, those using main push were more likely to receive high-level reading (OR = 4.867; 95% CI = 4.132-5.732) and re-sharing level (OR = 3.131; 95% CI = 2.706-3.623). Articles that featured exclamation/emphasis in the title attracted higher levels of user engagement (reading level: OR = 2.133; 95% CI = 1.826-2.491, re-sharing level: OR = 1.495; 95% CI = 1.289-1.735), while combinations of the above sentences in title type generally had low re-sharing level. Content, including other infectious diseases, chronic diseases, food safety and nutrition, vaccination, environmental and occupational health, health education activities, and healthy lifestyle, was associated with higher reading and re-sharing levels (*P* < 0.05) compared with other article contents. A combination of text, links, and pictures was associated with higher reading (OR = 3.530; 95% CI = 2.214-5.628) and re-sharing level (OR = 2.827; 95% CI = 1.791-4.462) compared with text alone. The greatest communication skills and marketing elements to promote the reading and re-sharing level were a combination of guidance/education/advice/appeal and negative emotional appeal (reading level: OR = 2.741; 95% CI = 2.205-3.408, re-sharing level: OR = 2.401; 95% CI = 1.950-2.956) and one marketing element (reading level: OR = 1.454; 95% CI = 1.258-1.681, re-sharing level: OR = 1.889; 95% CI = 1.643-2.172) compared to the reference, respectively. For article length, the greatest contribution to the reading and re-sharing level was 1000-1499 words (OR = 2.161; 95% CI = 1.858-2.513) compared with <1000 words, and closely followed by 1500-2000 words, while the least contribution was >2000 words. Articles with 1-149- second-, 150-300 second-, or >300-second-long videos were associated with a higher tendency of reading and re-sharing levels than no video.

**Figure 3:**
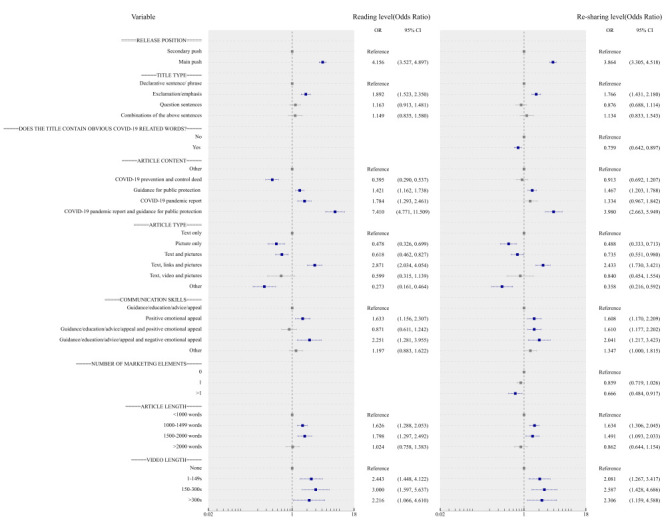
Multivariate logistic regression analysis of reading level and re-sharing level during COVID-19 outbreak period. s – seconds.

During the outbreak, release position, title type, and video length displayed a similar pattern with non-pandemic (**Figure 3**). However, pictures only (reading level: OR = 0.478; 95% CI = 0.326-0.699, re-sharing level: OR = 0.488; 95% CI = 0.333-0.713), and text and pictures (reading level: OR = 0.618; 95% CI = 0.462-0.827, re-sharing level: OR = 0.735; 95% CI = 0.551-0.980) were associated with a lower tendency of engagement behaviors compared to text alone. With the exception of a combination of guidance/education/advice/appeal and negative emotional appeal, positive emotional appeal also displayed a significant difference, receiving nearly 1.6 times as many likes as guidance/education/advice/appeal. Notably, posts that featured marketing elements (>1) (OR = 0.666, 95% CI = 0.484-0.917) or titles containing obvious COVID-19-related words (OR = 0.759; 95% CI = 0.642-0.897) attracted lower re-sharing levels. Additionally, articles with only 1000-1999 words were associated with higher reading and re-sharing levels. Nearly all articles from Chinese provincial CDCs during the outbreak were related to COVID-19 (>85%); consequently, we analyzed content characteristics related to COVID-19. The result showed that content about COVID-19 pandemic reports and guidance for public protection achieved the greatest contribution to reading (OR = 7.410; 95% CI = 4.771-11.509) and re-sharing levels (OR = 3.980; 95% CI = 2.663-5.949).

**Figure 4:**
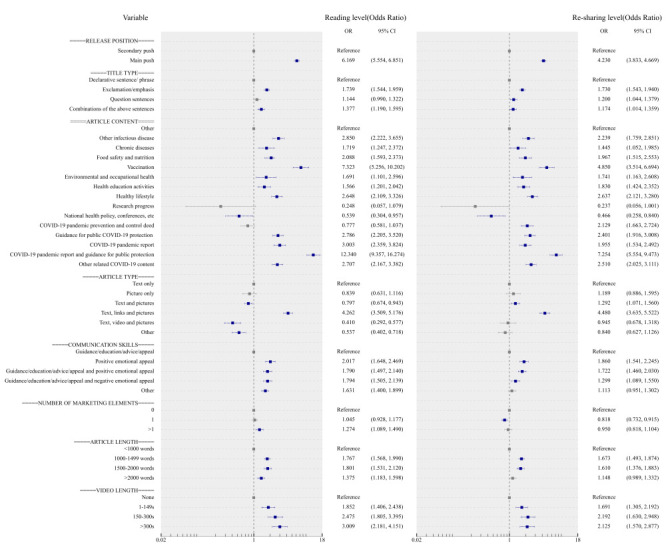
Multivariate logistic regression analysis of reading level and re-sharing level during COVID-19 normalization period. s – seconds.

During normalization, article features such as main push, a combination of exclamation/emphasis, positive emotional appeal, a combination of text, links, and pictures had the greatest contribution to reading and re-sharing levels compared to the control group (**Figure 4**). For article content, the public showed the greatest concern relating to COVID-19 pandemic reports and guidance for public protection (reading level: OR = 12.340; 95% CI = 9.357-16.274, re-sharing level: OR = 7.254; 95% CI = 5.554-9.473) and vaccination (reading level: OR = 7.323; 95% CI = 5.256-10.202, re-sharing level: OR = 4.850; 95% CI = 3.514-6.694). Content about national health policy, conferences, and other outlets were more likely to obtain low reading and re-sharing levels. Regarding marketing elements, a quantity greater than one attracted a high level of reading (OR = 1.274; 95% CI = 1.089-1.490). Articles containing 1500-2000 words were 1.801 and 1.610 times more likely to result in high-level reading and re-sharing than articles containing <1000 words.

### Predictive nomogram for the probability of high level of user engagement

**Figure 5:**
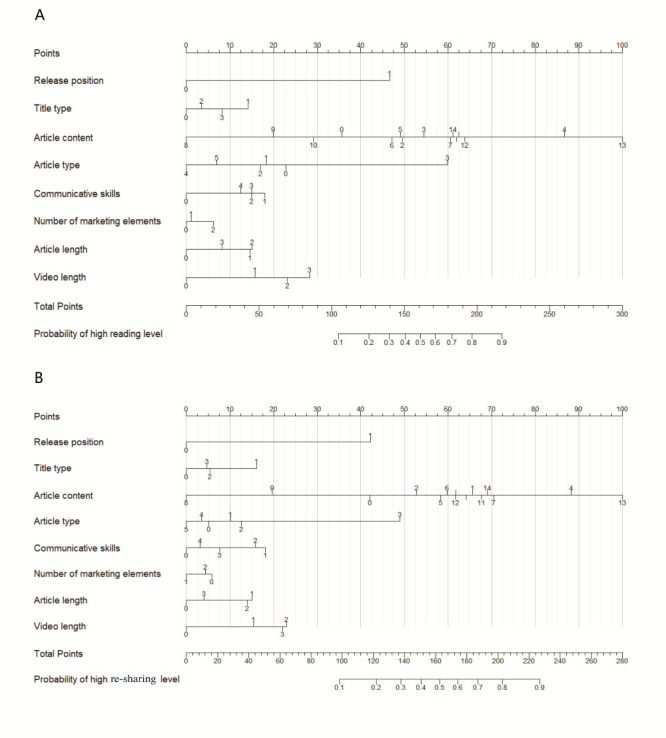
Nomogram predicts risk of user engagement for WeChat official accounts articles during COVID-19 normalization. **Panel A:** Nomogram predicts risk of reading level for WeChat official accounts articles. **Panel B:** Nomogram predicts risk of re-sharing level for WeChat official accounts articles. Release position (0: secondary push, 1: main push), title type (0: declarative sentence/phrase, 1: exclamation/emphasis, 2: question sentences, 3: combinations of the above sentences), article content(0: other, 1: other infectious disease, 2: chronic diseases, 3: food safety and nutrition, 4: vaccination, 5: environmental and occupational health, 6: health education activities, 7: healthy lifestyle, 8: research progress, 9: national health policy, conferences, etc.,10: COVID-19 pandemic prevention and control deed, 11: guidance for public COVID-19 protection, 12: COVID-19 pandemic report, 13: pandemic report and guidance for public protection, 14: other related COVID-19 content), article type (0: text only, 1: picture only, 2: text and pictures, 3: text, links, and pictures, 4: text, video, and pictures, 5: other), communication skills (0: guidance/education/advice/appeal, 1: positive emotion infection, 2: guidance/education/advice/appeal and positive emotional appeal, 3: guidance/education/advice/appeal and negative emotional appeal, 4: other), number of marketing elements(0: 0, 1: 1, 2: >1), article length(0: <1000 words, 1: 1000-1499 words, 2: 1500-2000 words, 3: >2000 words), video length (0: none, 1: 1-149 seconds, 2: 150-300 seconds, 3: >300 seconds).

Based on the final logistic regression analysis, we constructed a nomogram to predict article features associated with user engagement during normalization for the COVID-19 pandemic (**Figure 5**). The top three of eight key factors involved in article features at the reading and re-sharing level were the release position, article content, and article type. The calibration curve was in general agreement with the ideal curve and AUC was 81.6 (95% CI = 80.8-82.4) for the reading level and 77.9 (95% CI = 77.0-78.8) for the re-sharing level, indicating that the prediction model had good discriminatory power and calibration (Figure S1 in the **Online Supplementary Document**). We can search the corresponding score for the point scale axis of each article feature to gain the probability of a high level of user engagement during normalization for the COVID-19 pandemic.

## DISCUSSION

To the best of our knowledge, this is the first study to analyze Chinese provincial CDCs for WOAs article information generated at different pandemic stages and give insight into how those features have evolved and maximized user engagement during the COVID-19 pandemic. We found different feature patterns of articles at different pandemic stages and can thus provide valuable recommendations for government health agencies to use the best patterns to improve user online engagement to deal with public health emergencies in the future. Notably, article content, article type, and release position were the most prominent feature affecting article reading and re-sharing, indicating that the predominance of these features would be more appealing to readers.

We found that article content took priority over any other features during the COVID-19 pandemic. Significant topics, especially other infectious diseases, vaccinations, and healthy lifestyles had more positive effects on the number of readings and re-sharing than other topics, in line with a previous study [25]. Moreover, qualitative analyses and classifications of social media article content can provide important information for communications during a pandemic [28-31]. An analysis of Sina Weibo showed that domestic pandemics, quarantines, and investigations attracted more attention during the COVID-19 pandemic [32]. We also discovered that content including pandemic reports and guidance for public protection at the same time was most appealing to readers after the pandemic occurred. Additionally, vaccination has always been a priority at different periods, suggesting the importance of increasing content that the public is concerned about in the further.

Other than article content, another characteristic that significantly influenced the article's diffusion was article type. A combination of text, links and pictures has always been a priority over only text at different periods, possibly due to the effect of links. There is more controversy about the impact on links at the time of an emerging infectious disease [33]. For example, Xie et al. [23] received fewer re-shares during the COVID-19 pandemic, but Ngai et al. [34] reported a positive effect on sharing. Additionally, we found that pictures only or a combination of text and pictures during the outbreak period had a more negative effect on user engagement than only text, but other studies argued that articles with pictures have been re-shared with higher frequency [23,35]. However, emerging reports regarding the pandemic outbreak confirmed the positive effect of text-only articles [36,37]. This might be due to citizens paying more attention to an article’s textual content, rather than pictures in the face of public health emergencies [20], or to more pictures causing unnecessary consumption [38,39]. This is similar to the result from our study which showed that marketing elements, such as persons of authority or information sources, did not have a positive impact on user’ information behavior during a pandemic outbreak, suggesting that the association between article richness and user engagement should be further defined. The textual content has a positive impact on user' decision-making about article-reading and re-sharing behavior, but is inadequate for the period of the COVID-19 pandemic article’s rich elements should not be simply considered “the higher the better” and users might pay more attention to the text content itself during a pandemic.

Release position affected users’ information behavior concerning liking and re-sharing an article at any time, because enlarged cover pictures generated more traffic, suggesting important information should be put in the main push position. Attractive titles can engage more of the public [40] and our results also suggest the importance of an exclamation/emphasis title. We reported that titles containing obvious COVID-19-related words had a negative impact on users’ information behavior in terms of re-sharing an article during the COVID-19 outbreak, which was inconsistent with reports from the Guangzhou CDC [21]. This might be due to most articles during the outbreak being related to the COVID-19 pandemic and COVID-19-related titles not generating public attention.

For communication skills, before the pandemic occurred, a combination of guidance/education/advice/appeal and negative emotional appeal articles had a greater positive effect on user engagement than guidance/education/advice/appeal. As time progressed, emotions became more diverse [41]. During the outbreak, a combination of guidance/education/advice/appeal and negative emotions was most likely to promote citizen engagement, followed by positive emotional appeal. In agreement with reports on article content related to the pandemic [17], cancer [42], and so on, negative emotions lead to increase engagement. This finding might be due to articles with negative emotions article contents spreading faster, especially when facing to unexpected events [43,44]. When the pandemic gradually normalized, positive emotional articles also impacted public engagement behaviors online. Our result suggested that articles with emotions promoted citizen engagement behaviors, especially during the COVID-19 crisis.

WeChat provides a good channel for long articles sharing. Our results showed that articles with 1000-1499 and 1500-2000 words had a greater positive effect on user engagement than articles with 0-1000 words during any period, concluding that article length significantly affected more article’s diffusion [44,45]. Videos also had a positive effect on user engagement during any period, and optimal video length for user engagement was 150-300 seconds during the outbreak. This is because long texts or videos are likely to provide richer information than shorter ones [46].

However, we focused predominantly on the traditional dimension of citizen engagement, comprehensive engagement metrics should be explored to represent user engagement. Our result also might not apply to other social media platforms in other countries to respond to public health emergencies. We found that the determinants of users' behavior, including release position, title type, article content, article type, communicative skills, marketing elements, article length, and video length differed between pandemic stages. For example, when infectious diseases outbreak, article features with main push, exclamation/emphasis of title type, content focus on pandemic report and guidance for public protection, article type of text, links and pictures, guidance/education/advice/appeal and negative emotional appeal, 1000-1999 words, 150-300 seconds video length will attract more attention. We should encourage health sector to greater use of social media to reduce the burden of care on health with a focus on educating the public. These findings provide reference for government health agencies to improve the features of health information in the WeChat platform and engage their target audience during public health threats, allowing for a better grasp of information relevant to decreasing the threat of the pandemic [47].

## CONCLUSIONS

We found that determinants of users' behavior including release position, title type, article content, article type, communicative skills, marketing elements, article length and video length differed between pandemic stages. Notably, article content, article type, and release position were the most prominent feature affecting article dissemination, which could help public health agencies in choosing the best patterns to improve online user engagement when dealing with future public health emergencies.

## Additional material


Online Supplementary Document
