# Supplementary material for: Effects of Chinese provincial CDCs WeChat official account article features on user engagement during the COVID-19 pandemic
Source: J Glob Health. 2023 Apr 14;13:06005. doi: 10.7189/jogh.13.06005 (PMC10101727; doi:10.7189/jogh.13.06005)
Supplement: Online Supplementary Document [file jogh-13-06005-s001.pdf]

ONLINE SUPPLEMENTARY DOCUMENT

Title: Effects of Chinese Provincial CDC WeChat Official Accounts Tweets Features on User Engagement During the COVID-19 Epidemic

Authors: Mingjuan Yin, Senke Chen, Xueyan Pan, Caixia Lu, Xiaojie Lin, Mingwei Wang, Jindong Ni

Table S1. Variable Description

Item	Definition
**Ⅰ. Release position**	
Main push	The first of each push are headlines, following their card displays magnify the cover pictures.
Secondary push	Behind the main push among consecutive posted articles.
**Ⅱ. Title type**	
Declarative sentence/phrase	The sentence contains subject, predicate, and object, usually with a period, aiming to state a fact. Example: Novel Coronavirus vaccine safety and efficacy Authoritative explanation.
Exclamatory sentence or emphatic sentence	The sentence usually contains the subject, predicate, and object with an exclamation point, to express exclamation or emphasis and draw attention to. Example: These things will affect all doctors!
Question sentence	The sentence usually contains the subject, predicate, and object with a question mark. Sometimes without a question mark but express a question or rhetorical question. Example: Can the leftovers be eaten?
Combination of the above sentence	The title contains the above sentence patterns. Example: Who has sounded its highest public health alert! How is MONkeypox spread? How to prevent?
**Ⅲ. Content of the article relates to COVID-19**	
**Does the title contain obvious COVID-19 related words?**	
Yes	The title of the article contains words such as: COVID-19 / COVID-19 vaccine, epidemic/epidemic prevention, nucleic acid, case/confirmed/positive, etc.
No	Except the above.
**Article content**	
Epidemic report	Topic is related to reports for the number of new cases, dynamics of the epidemic, etc.
Guidance for Public Protection	Topic is related to vaccination, such as the correct

	way to wear a mask, wash hands to prevent COVID-19.
Basic knowledge of COVID-19	Topic is related to some professional knowledge about the COVID-19.
COVID-19 vaccine	Topic is related to vaccination, such as the introduction and precautions.
Prevention and control policies of COVID-19	Topic is related to documents, regulations and policies of the government agency.
COVID-19 prevention and control deed	Topic is related to advanced individuals or in Epidemic prevention and control.
Other	Content of the article is related to the unit meetings, work arrangements, etc.
**Ⅳ. Content of the article do not relate to COVID-19**	
Other Infectious disease	Topic is related to infectious diseases except COVID-19.
Chronic diseases	Topic is related to chronic diseases, such as diabetes, hypertension, etc.
Food safety and nutrition	Topic is related to food safety or nutrition. For example, the topic introduces food effects and gives recommendations.
Vaccination	Topic is related to vaccination except COVID-19 vaccine, such as the introduction and guidance of varicella vaccines.
Environmental and occupational health	Topic is related to air hygiene, drinking water hygiene, soil hygiene, housing hygiene, occupational diseases, etc.
Health education activities	Topic is related to events organized to conduct health education.
Healthy lifestyle	Topic is related to popular knowledge of life, such as how to lose weight, the benefits of drinking water, the dangers of staying up late, etc.
Scientific research progress	Topic is related to scientific research projects, including the laboratory construction of institutions.
National health policy, conferences, etc.	Topic is related to national meetings and policies related to the health industry, such as the National Health and Wellness Conference.
Other	Content of the article is related to the unit meetings, work arrangements, introduction of advanced deeds, etc.
**Ⅴ. Article type (multiple choices)**	
Texts	Article contains texts.
Videos	Article contains videos.
Pictures	Article contains pictures.
Links	Article contains links.

Other	Article contains other article types.
**Ⅵ. Communication skills (multiple choices)**	
Guidance / Education /Advice/ Appeal	Provide behavior guidance (eg. preventive control measures, etc.; Encourage users to take action (eg. call out, make an appointment, register for a show or event, etc.); Giving priority to instructive or informative information (including notices of free medical consultations, free medical examinations, lectures, etc.) in slogans texts; Call for forwarding; Appeal to a third party, etc.)
Questioning/Refute rumours	To question and correct popular opinions, statements made by "celebrities", or certain articles.
Positive emotional appeal	Aims to elicit positive emotions, such as using positive examples to convey hope and excitement.
Negative emotion appeal	Use some negative/negative cases to arouse users' fear or attract attention, such as listing the data of illness, etc. Use some negative cases to express criticism, such as the example of not cooperating with inspection and quarantine, punishment, etc.
Humor	Uses any humorous technique (such as sarcastic, jokes, etc.) to convey health messages, including funny pictures.
Other	Uses other the skills that do not mentioned above.
**Ⅶ. Marketing elements: (multiple choices)**	
Brand or sponsorship, partner	Any business, enterprise brand. Including any logos, colors, trademarks, slogans, related links, any activities supported by the brand (e.g. health campaigns, research activities, etc.) or other brands or services of brand partners.
Celebrities and sports people	Connecting people related to entertainment media or sports profiles with health events.
Films/Characters	Citing movies or characters, including characters from movies, books, TV shows and the Internet.
Persons of authority	Any person used for the purpose of lending their personal or positional authority to the health issue (eg. doctor, academic, scientist, politician).
Information sources	Citing sources of information such as books, guides, references, or instructions from other platforms.
APP or recommended by Official account	Appearing the QR code or link of some client applications to download or pay attention (excluding the current public account). Do not include advertisement at the end of the article.
Competitions, prizes, or giveaways	Any contest involving a participant entry, including

	minimal requirements such as liking or commenting on an article.
Dialogues	The article responds to readers' posts and comments, or quotes other online posts/news to share with members, including quoting offline conversations and interview records.
Texts	Collecting or testing participants' health knowledge.
Votes	Involving various types of voting for events or selections.
Other	Uses two and more of the skills mentioned above.
**Ⅷ. Article length**	
<1000 words	Amount of words in the article text.
1000-1499 words	Amount of words in the article text.
1500-2000 words	Amount of words in the article text.
>2000 words	Amount of words in the article text.
**Ⅸ. Video length**	Amount of second in the article video.

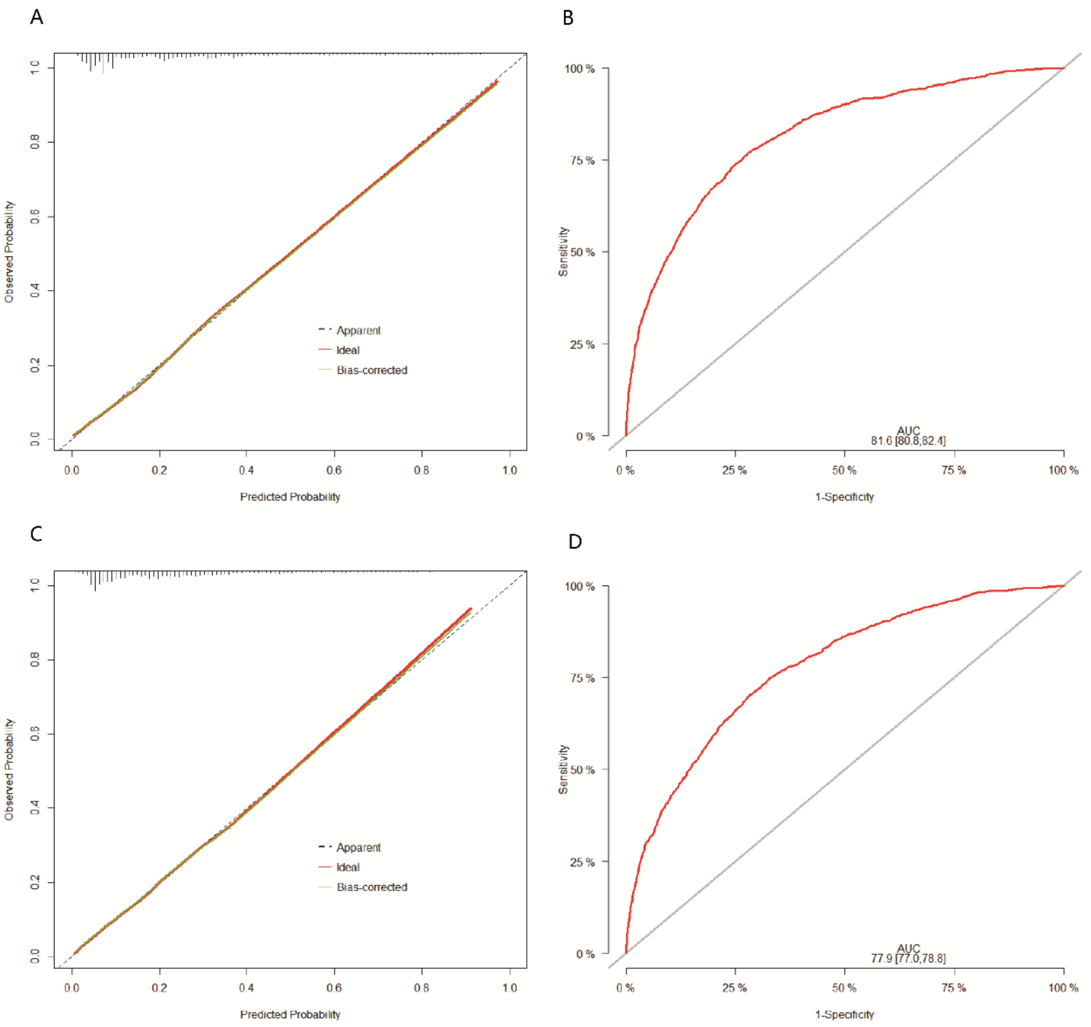

Figure S1. The receiver operating characteristic curves and the calibration plots of reading (AB) and retweet level (CD)
